# Accuracy of acute burns diagnosis made using smartphones and tablets: a questionnaire-based study among medical experts

**DOI:** 10.1186/s12873-017-0151-4

**Published:** 2017-12-13

**Authors:** Lisa Blom, Constance Boissin, Nikki Allorto, Lee Wallis, Marie Hasselberg, Lucie Laflamme

**Affiliations:** 1 Department of Public Health Sciences, Karolinska Institutet, Stockholm, Sweden; 2 Edendale Burn Services, Department of General Surgery, University of KwaZulu-Natal, Durban, South Africa; 3 Division of Emergency Medicine, Stellenbosch University, Bellville, South Africa; 4 University of South Africa, Institute for Social and Health Sciences, P.O. Box 1087, Lenasia, Johannesburg, 1820 South Africa

**Keywords:** Image-based diagnosis, Remote diagnosis, mHealth, Diagnostic accuracy, Burns, Handheld device, Smartphone, Tablet, Acute, Emergency

## Abstract

**Background:**

Remote assistance for burns by medical experts can support nurses and general physicians in emergency care with diagnostic and management advice. Previous studies indicate a high diagnostic accuracy based on images viewed on a computer screen, but whether image-based analysis by experts using handheld devices is accurate remains to be determined.

**Method:**

A review of patient data from eight emergency centres in the Western Cape, South Africa, revealed 10 typical cases of burns commonly seen in children and adults. A web-based questionnaire was created with 51 images of burns representing those cases. Burns specialists from two countries (South Africa and Sweden (*n* = 8 and 7 respectively)) and emergency medicine specialists from South Africa (*n* = 11) were contacted by email and asked to assess each burn’s total body surface area (TBSA) and depth using a smartphone or tablet. The accuracy and inter-rater reliability of the assessments were measured using intraclass correlation coefficients (ICC), both for all cases aggregated and for paediatric and adult burn cases separately. Eight participants repeated the questionnaire on a computer and intra-rater reliability was calculated.

**Results:**

The assessments of TBSA are of high accuracy all specialists aggregated (ICC = 0.82 overall and 0.81 for both child and adult cases separately) and remain high for all three participant groups separately. The burn depth assessments have low accuracy all specialists aggregated, with ICCs of 0.53 overall, 0.61 for child and 0.46 for adult cases. The most accurate assessments of depth are among South African burns specialists (reaching acceptable for child cases); the other two groups’ ICCs are low in all instances. Computer-based assessments were similar to those made on handheld devices.

**Conclusion:**

As was the case for computer-based studies, burns images viewed on handheld devices may be a suitable means of seeking expert advice even with limited additional information when it comes to burn size but less so in the case of burn depth. Familiarity with the type of cases presented could facilitate image-based diagnosis of depth.

**Electronic supplementary material:**

The online version of this article (10.1186/s12873-017-0151-4) contains supplementary material, which is available to authorized users.

## Background

Burns are disproportionally concentrated to low-income settings in general and Sub-Saharan Africa in particular [1, 2]. Emergency care services in these settings are often less equipped to deal with the problem, as they are dealing with a wide range of health problems with limited training and support. Diagnosis is one important component in providing appropriate emergency care [3], not least in the case of burns, where the acute phase is crucial for treatment outcome [4], the severity assessments at point-of-care are often inaccurate [5–8], and specialised assistance from burn centres is often limited. Inaccurate diagnosis leads to under- or over- triage, management errors in fluid calculations [7, 8] and potentially to problems in decisions on airway management [5].

Telemedicine – and more specifically mHealth – can help to provide timely burn diagnosis support at point-of-care [9–11]. A prior study showed that image-based burn severity assessment by experts is accurate when the images are viewed on a computer screen [12]. Similar results have been reported for specialities like dermatology [13, 14], radiology [15, 16] and ophthalmology [17]. Nowadays however, with the wide availability and ownership of handheld devices like smartphones and tablets, physicians are more inclined to use their private smartphones rather than their computers for work-related communications, not least when it comes to seeking and providing image-based advice [18, 19]. Informal communication using mobile phones, for example through WhatsApp, is increasingly used for remote diagnosis [19, 20], and is becoming common practice for acute burns consultation in South Africa [21, 22]. Studies are lacking however as to how accurate assessments made this way are. As handheld devices offer more flexibility than computers, allowing experts to review cases on their own handheld device would be timesaving, which is an important asset in the emergency care environment. In terms of image quality (disregarding the diagnostic accuracy), there is evidence that images of various kinds viewed by medical staff on handheld devices are perceived as being of comparable quality as when viewed on computer screens [23, 24]. Also, in fields like radiology and echocardiography it has been reported that experts can accurately diagnose images on tablets [25, 26] and smartphones [27, 28]. Assessments of minor burns (0.1–5% total body surface area - TBSA) were made with similar accuracy as live assessments using an early generation of smartphones [29]. Such evidence is lacking when it comes to a wider range of burns severity, and also when including both smartphones and tablets. This study was embarked upon to address this knowledge gap. The following research questions were addressed: How accurate is the image-based remote diagnosis of burns commonly presenting to emergency centres of the Western Cape, when viewed on a handheld device? Are remote assessments of comparable accuracy when made on handheld devices compared to a computer screen?

## Methods

The study involved 51 images representing 10 typical burns cases that are commonly seen at emergency centres, viewed by 26 burns and emergency medicine experts on handheld devices. Eight of the participants also conducted the survey on a laptop computer.

### Case and image selection

The cases presented in the survey were selected in three steps, as described below.

#### Identification of typical injury cases from a one-year caseload

Typical injury cases were identified from a one-year caseload (*n* = 1913) of burn patients in eight emergency centres [30] in the province. In this patient group, 39.4% were 0–4 years, 10.2% were 5–9 years and 28% were 20–39 years. Slightly more than half were men (52.8%) and about two thirds (65.2%) were burnt by hot liquids [30]. For the purpose of this study, the dataset was first split into two age groups: children (0–12 years) and adults (13 years and older), on grounds that paediatric burns units (as other healthcare services) treat children 0–12 years while those 13 years and above are treated in adults units [31]. Then, for those two categories, the body part burned was used and five “typical cases” were selected based on the most common body parts burned for children and adults (Table 1).

**Table 1:** Typical cases of burns patients at emergency centres in the Western Cape

Body part	Children (*n* = 1013) % of cases	Adults (*n* = 900) % of cases
Lower extremities	13.1	13.9
Trunk including buttocks	12.1	11.3
Upper extremities excluding hands	9.6	9.9
Hands	8.6	–
Upper extremities and trunk	–	7.7
Head	7.9	7.1

#### Selection of related images

Thereafter, pictures gathered from a bank of burn images were reviewed and sorted into these cases. The image had to exclusively reflect the full body part assigned to it. One patient could be represented in several cases if the patient had burns on multiple body parts since images often are captured by physicians focused on one body part. For each of the 10 cases, 5–10 burn images were selected to have a variety of burns within each case based on injury parameters of importance for visual assessment (size, depth and mechanism).

#### Consultation with local emergency physician for representativeness

The selection was reviewed by a physician working in one of the emergency centres (who did not participate in the study) to confirm representativeness of the patient group that are seen at emergency centres in the province and appropriateness for inclusion. In the final selection, 5–6 images were chosen for each case, resulting in 51 images to assess in total. Table 2 presents the main characteristics of the patients and injuries under study. Child cases had a range of 0.5–12% TBSA, while the range of adult cases was 2–23% TBSA. Both child and adult cases had depths ranging from superficial partial thickness to full thickness.

**Table 2:** Patient and injury characteristics of the images included expressed in numbers or range^a^

Characteristics	Children (*n* = 25)	Adults (*n* = 26)
Mean age in years (range)	2.9 (0–9)	31.5 (19–59)
Male/female	9/16	18/8
Fitzpatrick skin type (range)	4–6	2–6
Mechanism		
Hot liquid	17	9
Fire	3	16
Contact	4	–
Electric	1	–
Unknown	–	1
TBSA range	0.5–12	2–23
Depth range	Superficial partial – full	Superficial partial – full

^a^of the total of 51 images, four were of different body parts of the same two children and 12 were of different body parts of four adults

### Participants

A sample of clinical experts was purposively enrolled with the requirement of having a competence in diagnosing burns, either by training or by clinical practice. The participants were contacted through one of the following three groups: 1) the physicians enrolled as tele-experts in the mHealth project that the research team is involved in [32] (burn specialists *n* = 2 and emergency medicine specialists *n* = 11); 2) burns specialists from Sweden that have shown an interest for this field of research and are themselves involved in related studies (*n* = 7); 3) burns specialists from other provinces belonging to the network of the tele-experts in the first group (*n* = 6). We assigned the participants into three groups based on their speciality and country of practice; South African emergency medicine (EM) specialists (n = 11), South African burns specialists (*n* = 8) and Swedish burns specialists (n = 7).

### Data collection instrument

**Fig. 1:**
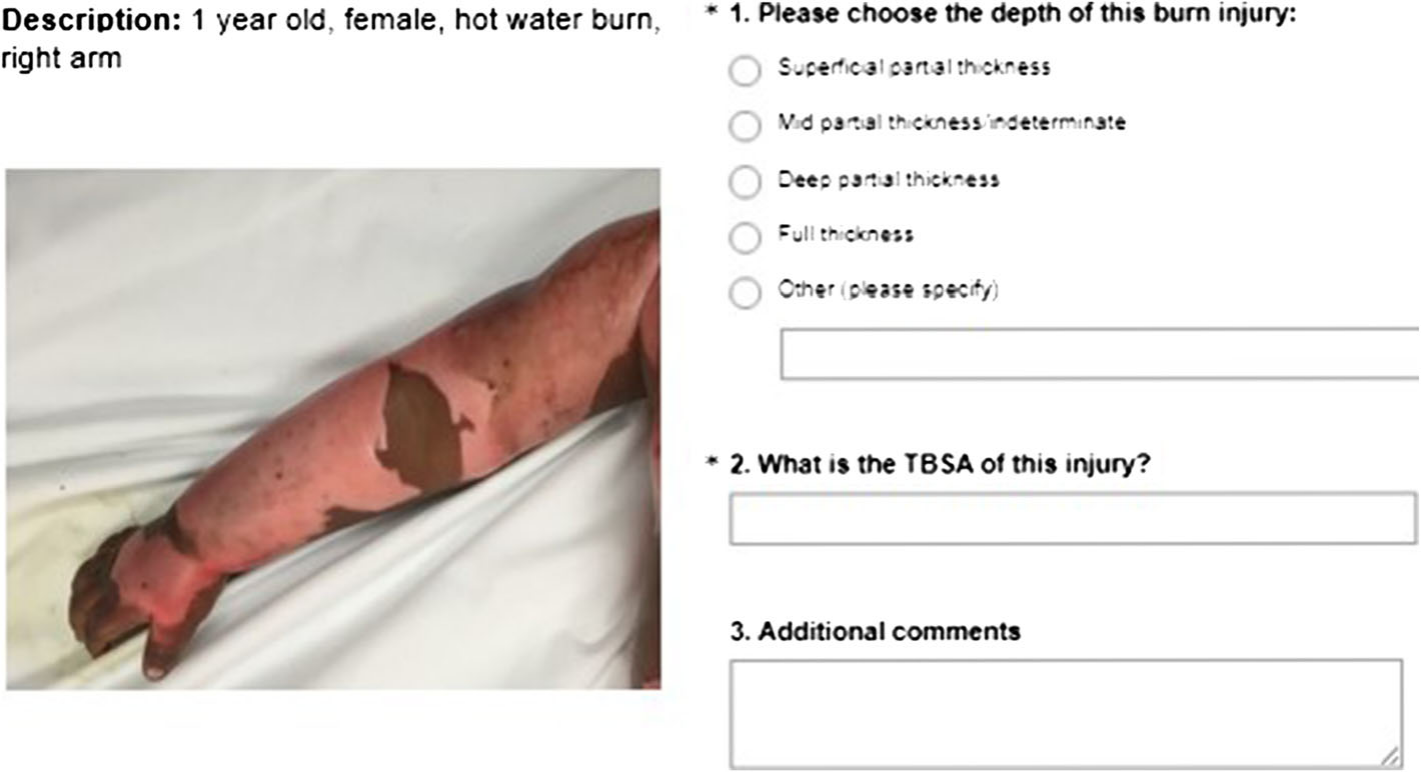
Example of an image and the descriptive information presented to the participants

A web-based questionnaire was developed using SurveyMonkey™. The questionnaire started with questions on participants’ background, demographics, prior experience of remote consultations and type of device used (smartphone or tablet). Thereafter, the 51 images were presented along with a brief description (age, sex and mechanism of injury) (Fig. 1). The participants were asked to assess burn depth (four categories: superficial partial, mid partial/indeterminate, deep partial, and full thickness [33]) and size, expressed as TBSA (in percentage, free text). Additional comments could be made separately about each image. The questionnaire ended with a few final questions about the participants’ confidence when making a diagnosis based on the images in the questionnaire, if they would feel comfortable advising in a telemedicine system with similar images and whether they perceive images as helpful in making a diagnosis.

### Data collection procedure

The first and second author contacted eligible participants via email with an information letter introducing the aim and objective of the study. A link to the SurveyMonkey questionnaire was sent in the email and they were asked to reply to this questionnaire using a handheld device of their choice (smartphone or tablet). The images were presented in a randomised order. Participants were allowed to complete the survey on different occasions by using the same login and resuming working from where they ended on the last entry.

Eight participants were asked to answer the questionnaire again on a laptop computer (minimum two weeks after the first session) at a later time. This was done on a voluntary basis (convenience sampling) and we asked only those respondents who could have access to the computer specifically used for this part of the study. All three groups of clinicians were represented among those who repeated the survey. They all agreed to this and were provided with the computer that was designated for the purpose with a fixed screen resolution and luminance (HP EliteBook 1040 G3 256 SSD with the screen: 14 in., 1920 × 1080 resolution). The images were presented to them in random order and the participants were given the possibility to complete the survey at their own pace in a quiet place.

### Statistical analyses

Real time bedside assessment was considered the gold standard for both TBSA and burn depth. Diagnostic accuracy was assessed versus gold standard using a two-way mixed effect intraclass correlation coefficient (ICC) for both TBSA [34] and burn depth (as an equivalent for weighted kappa [35]). Six answers for TBSA (0.5%) and 27 for depth (2%) out of 1326 were left unanswered. For the accuracy analyses, these were treated as missing and removed. ICC was also used for inter-rater reliability of participants’ diagnoses of burn depth and size, and for intra-rater reliability between those participants that conducted the survey twice. For TBSA, the standard error of measurement (SEM) was calculated. For these reliability analyses, the missing answers were replaced by the answer that deviated most from the gold standard for that specific image. For TBSA this was based on the answer of another participant; for depth, the most distant category was used. Analysis was performed for all images combined, as well as stratified on patient age (children/adults) and speciality and country of practice of participants (three categories). A confidence interval (CI) of 95% was used for all ICCs.

The interpretation of ICC by de Vet [36] used by Hop et al. [37] for photographic assessment of burn size and depth was applied and is as follows:

$$ <0.70=\mathrm{low} $$

$$ 0.70\hbox{--} 0.80=\mathrm{acceptable} $$

$$ >0.80=\mathrm{high} $$

A Bland-Altman plot was created to illustrate the differences in assessments of TBSA compared to the gold standard of each image. To measure the sensitivity and specificity of the assessments of depth, cases were dichotomised based on whether one could expect that surgery would be required (deep partial and full thickness) or not (superficial partial or mid partial thickness/indeterminate). Sensitivity was compiled by calculating the proportion of those burns with a gold standard as either deep partial or full thickness that were assessed correctly by the participants. Specificity was compiled by the proportion of the burns with a gold standard as superficial partial or mid partial/indeterminate thickness that were assessed correctly. Missing answers were excluded from both the Bland-Altman plot and the sensitivity analysis of depth. Stata/IC 12.1 for Windows was used for all analyses [38].

## Results

Table 3 presents some information about the participants stratified by specialty and country. In total, the participants have a mean age of 41 years (range 29–75 years) and eight of the 26 participants are women. More than half of the participants (15 of 26) use handheld devices for image-based assessments at least a few times a week and this varied according to country of practice with it being more common among the South African participants (6 of 11 emergency medicine specialists and 7 of 8 South African burns specialists) compared to the Swedish burns specialists (2 of 7).

**Table 3:** Information about the participants by country of practice and domain of speciality

Country	All	South Africa		Sweden
Demographics and use of device	Total n = 26	EM n = 11	Burns n = 8	Burns n = 7
Age (in years)				
Mean (range)	40.9 (29–75)	37.5 (31–47)	41.1 (29–75)	46.1 (30–64)
Gender				
Female, n	8	1	5	2
Male, n	18	10	3	5
Use of handheld device for image-based assessments				
A few times a week or more	15	6	7	2

Table 4 shows the accuracy and inter-rater reliability for results of TBSA. The accuracy of image-based assessments of TBSA on handheld devices is high with an ICC of 0.82 (95% CI 0.81–0.84) overall and of 0.81 for both child (95% CI 0.78–0.83) and adult (95% CI 0.78–0.84) cases separately. The inter-rater reliability of these assessments is high overall (0.81; 95% CI 0.74–0.87; SEM of 2.59%) and in child cases (0.81; 95% CI 0.72–0.89; SEM of 1.95%) and acceptable in adult cases (0.78; 95% CI 0.68–0.88; SEM of 3.01%).

**Table 4:** Diagnostic accuracy and inter-rater reliability of TBSA assessments made on handheld devices

Cases	Accuracy^a^	Inter-rater reliability
	ICC (95% CI)	ICC (95% CI)
Overall	0.82 (0.81–0.84)	0.81 (0.74–0.87)
Children	0.81 (0.78–0.83)	0.78 (0.68–0.88)
Adults	0.81 (0.78–0.84)	0.81 (0.72–0.89)

^a^6 missing values, analysis performed on 1320 cases

When stratifying by speciality and country of the participants, the accuracy of the assessments of TBSA remain high for all groups (Table 5) but slightly lower for South African emergency medicine specialists (0.80; 95% CI 0.77–0.83) than for burns specialists among either South Africans (0.87; 95% CI 0.85–0.89) or Swedes (0.87; 95% CI 0.84–0.89). For children, the accuracy was somewhat higher for South African burns specialists (0.90; 95% CI 0.87–0.92) than South African emergency medicine specialists (0.77; 95% CI 0.72–0.81) while the assessments of adult cases were relatively similar between the three groups. The inter-rater reliability of TBSA is high overall and comparable among the three groups with overlapping CIs. The three participant groups had acceptable to high inter-rater reliability for both child and adult cases.

**Table 5:** Diagnostic accuracy and inter-rater reliability of TBSA assessments made on handheld devices by participant group

Cases	Participants	Accuracy^a^	Inter-rater reliability
		ICC (95% CI)	ICC (95% CI)
Overall	South African EM specialists	0.80 (0.77–0.83)	0.81 (0.74–0.87)
	South African burns specialists	0.87 (0.85–0.89)	0.81 (0.74–0.87)
	Swedish burns specialists	0.87 (0.84–0.89)	0.85 (0.79–0.90)
Children	South African EM specialists	0.77 (0.72–0.81)	0.75 (0.64–0.86)
	South African burns specialists	0.90 (0.87–0.92)	0.85 (0.77–0.92)
	Swedish burns specialists	0.83 (0.78–0.87)	0.76 (0.64–0.87)
Adults	South African EM specialists	0.79 (0.75–0.83)	0.81 (0.71–0.89)
	South African burns specialists	0.85 (0.81–0.89)	0.79 (0.68–0.88)
	Swedish burns specialists	0.87 (0.83–0.90)	0.88 (0.80–0.93)

^a^6 missing values, analysis performed on 1320 cases

**Fig. 2:**
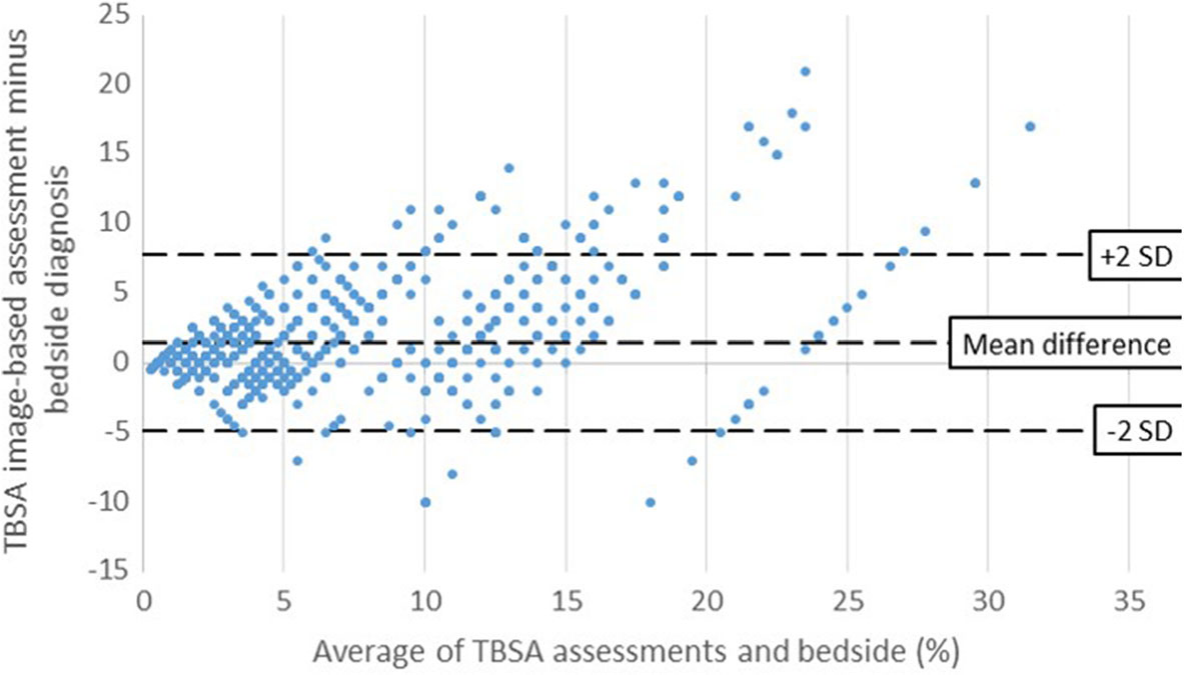
Bland-Altman plot of the TBSA assessments minus bedside diagnosis against the mean TBSA per image

Figure 2 shows that participants both under- and over-estimated the burns, although overestimations were more common. The range of the differences were greater among larger burns.

The accuracy of the ratings made on handheld devices for burn depth (Table 6) is low overall but slightly higher in child cases than in adult cases. The inter-rater reliability of burn depth is low with ICC values of 0.43 (95% CI 0.34–0.54) overall, and of 0.51 (95% CI 0.38–0.67) and 0.36 (95% CI 0.24–0.53) for child and adult cases respectively.

**Table 6:** Diagnostic accuracy and inter-rater reliability of depth assessments made on handheld devices

Cases	Accuracy^a^	Inter-rater reliability
	ICC (95% CI)	ICC (95% CI)
Overall	0.53 (0.49–0.57)	0.43 (0.34–0.54)
Children	0.61 (0.55–0.65)	0.51 (0.38–0.67)
Adults	0.46 (0.40–0.52)	0.36 (0.24–0.53)

^a^27 missing values, analysis performed on 1299 cases

The accuracy of the assessments of depth made by South African burns specialists overall and in children are significantly higher than those of South African emergency medicine specialists and higher than those of Swedish burns specialists, although the results are not significant (Table 7). For adults, the accuracy is low among all three groups and the CIs overlap. The inter-rater reliability of assessments of depth among the three participant groups are low in all instances and the CIs overlap. Analysis of the assessments of depth corresponding to burns that would require surgery results in 75.7% sensitivity and 70.4% specificity.

**Table 7:** Diagnostic accuracy and inter-rater reliability of depth assessments made on handheld devices by participant group

Cases	Participants	Accuracy^a^	Inter-rater reliability
		ICC (95% CI)	ICC (95% CI)
Overall	South African EM specialists	0.49 (0.43–0.55)	0.43 (0.33–0.55)
	South African burns specialists	0.64 (0.58–0.69)	0.50 (0.39–0.62)
	Swedish burns specialists	0.51 (0.43–0.59)	0.49 (0.38–0.62)
Children	South African EM specialists	0.54 (0.45–0.62)	0.46 (0.32–0.64)
	South African burns specialists	0.75 (0.68–0.80)	0.63 (0.48–0.78)
	Swedish burns specialists	0.59 (0.48–0.68)	0.49 (0.33–0.68)
Adults	South African EM specialists	0.44 (0.35–0.53)	0.41 (0.27–0.59)
	South African burns specialists	0.54 (0.43–0.63)	0.39 (0.24–0.57)
	Swedish burns specialists	0.45 (0.33–0.56)	0.50 (0.34–0.68)

^a^27 missing values, analysis performed on 1299 cases

The accuracy of the assessments of TBSA made on a computer screen (Table 8) are high overall (0.85; 95% CI 0.82–0.88), in child (0.90; 95% CI 0.87–0.92) and in adult cases (0.82; 95% CI 0.77–0.86). The participants’ assessments of TBSA made on handheld device and computer are similar with results indicating high intra-rater reliability (0.88, 0.85 and 0.87 overall and in child and adult cases respectively).

**Table 8:** Diagnostic accuracy and intra-rater reliability (handheld vs computer) of TBSA assessments made on computer

Cases	Accuracy	Intra-rater reliability
	ICC (95% CI)	ICC (95% CI)
Overall	0.85 (0.82–0.88)	0.88 (0.85–0.90)
Children	0.90 (0.87–0.92)	0.85 (0.80–0.88)
Adults	0.82 (0.77–0.86)	0.87 (0.84–0.90)

The accuracy of burn depth assessments made on a computer screen (Table 9) are low with ICC of 0.48 overall (95% CI 0.41–0.55), 0.50 for child burns (95% CI 0.39–0.60) and 0.46 for adult burns (95% CI 0.35–0.56). Intra-rater reliability between the assessments made on handheld device and computer is also low (0.63, 0.65 and 0.62 overall and in child and adult cases respectively).

**Table 9:** Diagnostic accuracy and intra-rater reliability (handheld vs computer) of depth assessments made on computer

Cases	Accuracy	Intra-rater reliability
	ICC (95% CI)	ICC (95% CI)
Overall	0.48 (0.41–0.55)	0.63 (0.57–0.69)
Children	0.50 (0.39–0.60)	0.65 (0.56–0.72)
Adults	0.46 (0.35–0.56)	0.62 (0.52–0.69)

About half of the participants stated that they felt mostly (*n* = 9) or completely (*n* = 2) confident in making a diagnosis from the images in the survey and the remaining 15 said that confidence varied depending on the case. More individuals among the burns specialists from both South Africa (6 of 8) and Sweden (3 of 7) stated that they felt confident than among the South African emergency medicine specialists (2 of 11). As many as 24 of the 26 participants stated that they would feel somewhat (*n* = 15) or completely (n = 9) comfortable to use a telemedicine system for burns using similar images and information. All participants affirmed that images are helpful for diagnosing and providing advice in burn care.

## Discussion

### Main findings

Using handheld devices for image-based advice among clinicians is spreading rapidly in a range of clinical settings [20, 21, 39–41], but the practice lacks solid evidence as regards diagnostic accuracy. To the best of our knowledge, this is the first scientific study to investigate this question more closely, with a focus on acute burns, i.e. a type of injury that challenges clinicians in emergency care services. By and large, we observed that the results concerning burn size are somewhat better than those relative to burn depth, just as is the case for assessments on computer screens [12].

We found that, in all three participant groups, the assessments of burn TBSA was high for all cases aggregated and for child and adult cases separately. In addition, compared to when using a computer, the assessments on handheld devices were of comparable accuracy. The results are similar to those from prior studies [29, 37, 42]. With regard to clinical expertise, the burns specialists in our study had the same ICC as the burns specialists in an earlier study [37].

In the case of burn depth, which is challenging even at bedside [43], the assessments were less accurate. South African burns specialists were slightly more accurate than the other two groups and the assessments of paediatric cases were better than those of adults. In addition, there was not a remarkable difference between making an assessment on a handheld device than on a computer screen. It is of note however, that these results were considerably better than those from one study using photographs of patients [37] but poorer than another [42]. The finding that assessments of depth among South Africans were more accurate among burns specialists (overall and in children) than emergency medicine specialists is most probably a reflection of differences in training and experience. Another plausible explanation could be that TBSA is more important for initial assessment at the emergency phase to work out resuscitation requirements, while depth has greater implication when assessing the need for surgery, which involves burns specialists to a greater extent. This point was raised by some of the participants in their free text comments.

There are mixed results as regards under- and over-estimation of burns severity. When considering whether the patients would require surgery or not according to gold standard of depth, three out of four burns that were deep partial or full thickness (75.7%), and almost the same proportion that were superficial partial or mid partial/indeterminate thickness (70.4%) were assessed accordingly on handheld devices. This finding suggests that overestimation of depth occurs in similar proportions as underestimation among the participants. These results differ from an investigation by Hop et al. [37], where overestimation was more common with reported 66.8–83.8% sensitivity and 40.9–46.2% specificity.

### Strengths and limitations

This study contributes new knowledge on the potential accuracy of remote assessments of acute burns made on handheld devices by experts which is, as mentioned above, a practice on the increase and rapidly substituting laptop screens. A few elements making the study more robust are worth mentioning. To start with, the design was such that images were presented in random order to avoid any order bias and the participants were given the possibility to use a handheld device of their choice. The latter gave a sense of real-life setting but it is of note that the use of smartphones was far more common than that of tablets. An additional design-related strength is that, for the intra-rater reliability, the time lag between the two measurements (minimum two weeks but many times longer than that) was most certainly sufficient to avoid memory bias given the length of the questionnaire and the high number of images to assess. The high number of images included was a way to make the study more robust, as well as having all participants assessing all images. In line with this, answering the questionnaire was relatively time-consuming but the participants could take a break and resume to allow for flexibility and limit the risk of response fatigue. The questionnaire had a low number of missing answers and different methods were tested in how to treat these but neither of them affected the results considerably.

One limitation of note is the fact that on some images there were several burn areas with different depths. Whereas it was important to include these since it represents the reality of emergency care situations, images of burns with one depth only would have been more straightforward for assessment and analyses purposes. Expecting some difficulties in that respect, the participants were instructed to choose only one depth for each image and to focus on the proportionally predominant depth. However, for 15 images, the proportions were relatively similar, which puts the participants in a challenging situation. Low agreement has been reported earlier when images include a mixture of depths [42]. Although the number of images with mixed depths herein was relatively small, it is possible that it could have lowered the agreement. Regarding the sensitivity analysis of burn depth, actual data on surgery status was not available and therefore the depth categories are only serving as an indicator for potential need of surgery. Bedside assessment was used as the gold standard rather than other techniques such as the laser Doppler [44] since these are not in practice in burn care in South Africa. An additional limitation is that the setup of our study differs slightly from clinical practice where an image sent for consultation often comes with more information than what was provided herein, for example more detailed patient and injury characteristics as well as the opportunity to chat or follow up on phone. This means that our experts were forced to make assessments under less than optimal circumstances. This was also a point that was raised in the free text comments by the participants. There is therefore the possibility that the results are on the lower side of the spectrum and that assessments made in clinical practice could be even more accurate. A third potential limitation is that a larger sample of images would be needed to allow for more detailed stratification in order to provide more information about the characteristics of cases that are particularly challenging to assess. A total of 50 images have been recommended for sample size of validation studies [36] but for stratification by typical cases, a larger number of images would have been needed. This was considered when designing the study but the number of images was limited by the time it would require in terms of dedication from each participant. The mixed-effect ICC limits the potential for generalisability since it is a model that assumes that the participants are not a random sample of a larger population [34]. This model was considered appropriate since all participants reviewed all images, and the participants were selected purposely. This also means that the representability of the results on the participants’ country and specialty could be debated due to the small selection of participants from each country. It is of note however that in any specific country, the number of burns specialists is relatively low. Lastly, there is some imbalance between the participants in terms of demographics and practice of using handheld devices for image-based remote assessments. Being more used to working with smartphones and tablets could also facilitate making a diagnosis through these devices.

### Implications

With the increasing use of handheld devices for remote assessments [20–22, 39–41], knowledge of the accuracy of diagnosing through this means is necessary. This study provides encouraging evidence on the applicability of, and the positive attitude among participants towards using smartphones and tablets for diagnostic support on burns. The levels of accuracy observed may represent under-estimation of the accuracy that could be reached in more “real practice” situations. Indeed, in this study, the participants were only provided with minimal information on the cases they had to assess but in several systems under development or implementation, chat functions can be used that can work as a good complement [32].

Whether some types of burns are more challenging than others – or for some particular groups of assessors – is also a question that deserves additional consideration in future studies. Finally, given the increasing use of the practice and the potential importance of familiarity with the type of cases to be assessed for the accuracy of the diagnosis set, providing specialists with training on image-based diagnosis could be beneficial. On the other hand, the satisfactory results of the Swedish participants indicate that remote advice could also be provided by non-local specialists.

## Conclusion

As was the case for computer-based studies, burns images viewed on handheld devices may be a suitable means of seeking expert advice even with limited additional information when it comes to burn size but less so in the case of burn depth. Given the flexibity of the smartphone applications available nowadays to clinicians, which allow for images to be sent with additional information and to chat between clinicians, it is likely that our results, based in the main on images only, are on the conservative side and assessments in clinical practice are likely to be more accurate.

When considering country of practice and specialty, levels of accuracy varied somewhat more for burn depth than size and the burns specialists from South Africa had conspicuously better results, most likely because of their familiarity with the type of cases the participants were presented with.

## Additional file

Additional file 1: Raw data for Table S4 and S6. The file contains raw data for Tables S4 and S6 with assessments of TBSA and depth for each image and participant. Participant 1 is the bedside diagnosis which is used as the gold standard for all analyses. Participant 1 was not used for reliability analyses. (XLSX 42 kb)

## Abbreviations

CI: Confidence interval
EM: Emergency medicine
ICC: Intraclass correlation coefficients
SEM: Standard error of measurement
TBSA: Total body surface area
